# Understanding PFAS: Occurrence, Fate, Removal, and Effects

**DOI:** 10.3390/toxics12080605

**Published:** 2024-08-19

**Authors:** Chang-Gui Pan, Run-Xia Sun

**Affiliations:** 1 Guangxi Laboratory on the Study of Coral Reefs in the South China Sea, School of Marine Sciences, Guangxi University, Nanning 530004, China; 2 School of Marine Sciences, Nanjing University of Information Science and Technology, Nanjing 210044, China; sunrunxia@nuist.edu.cn

Per- and polyfluoroalkyl substances (PFASs) have emerged as one of the most pressing environmental and public health concerns of the 21st century. PFASs are a large group of humanmade chemicals characterized by their carbon–fluorine bonds, which are among the strongest in organic chemistry [1]. This bond imparts remarkable stability to PFASs, making them resistant to environmental degradation processes such as hydrolysis, photolysis, and microbial degradation [2]. Due to their water- and oil-repellent properties, PFASs have been widely used in various industrial and consumer products, including in firefighting foams, stain-resistant fabrics, and non-stick cookware, etc. [3]. The wide application, persistence, and bioaccumulative properties of PFASs have led to their widespread contamination in water, sediment, air, living organisms, and even in remote areas [4], which underscores their global distribution. In addition, the issue of PFASs is characterized by their diverse chemical formulations, and not all PFASs behave identically in the environment. For example, representative PFASs, such as perfluorooctanoic acid (PFOA) and perfluorooctane sulfonate (PFOS), are particularly persistent and have been detected at relatively high concentrations [5]. Understanding the fate of PFASs in the environment is essential for assessing their long-term impact and developing effective remediation strategies. Moreover, PFASs have been associated with a range of health issues, including cancer, liver damage, immune system disruption, and developmental effects [6]. As a result, their environmental ubiquity, persistence, bioaccumulation, and potential toxic health effects (PBT) have galvanized scientific research, policy discussions, and public concern.

In this context, the articles published in this Special Issue highlight the occurrence, fate, and effects of PFASs. Several articles explore the complexities of pollution assessment, offering insights into the impact of pollutants on environmental health and human wellbeing. Studies on degradation processes underscore the importance of understanding the ecological consequences of pollution. Consequently, a key theme of these investigations is the urgent need for effective mitigation measures. Additionally, the articles offer valuable guidance for policymakers, practitioners, and researchers.

As academic editors for this Special Issue, we are especially excited by the diverse topics covered in this collection. We hope that the discoveries presented will inspire further interdisciplinary collaboration and innovative solutions to the challenges of environmental pollution. The complexity of this Special Issue lies in interpreting the occurrence, fate, removal, effects, and management strategies of PFASs.

The paper “In Vitro and In Silico Analysis of the Bindings between Legacy and Novel Per- and Polyfluoroalkyl Substances and Human Serum Albumin” examined the binding mechanisms of human serum albumin (HSA) to legacy PFASs and their novel alternatives using fluorescence spectroscopy and molecular docking. Their results showed that all target PFASs statically quenched HSA with binding ratios of 1:1. Long-chain PFASs and novel perfluoroalkanesulfonic acids (PFSAs) had binding constants greater than 10^2^, while short-chain PFASs and novel perfluorocarboxylic acids (PFCAs) had binding constants less than 10^2^. Generally, PFCAs had lower binding affinities to HSA than PFSAs, and short-chain PFASs had lower affinities than long-chain PFASs and their novel alternatives. Thus, binding to HSA is a key factor in the bioaccumulation of legacy and novel PFASs in the human body.

The paper “Biodegradation Potential of C7-C10 Perfluorocarboxylic Acids and Data from the Genome of a New Strain of *Pseudomonas mosselii* 5(3)” explored the ability of *Pseudomonas mosselii* strain 5(3), isolated from pesticide-contaminated soil, to degrade C7-C10 PFCAs and analyzed its complete genome. The genome consists of a chromosome 5,676,241 base pairs long with 5134 genes, including those for haloalkane dehalogenase (*dhaA*), haloacetate dehalogenase H-1 (*dehH1*), fluoride ion transporter (*crcB*), and alkanesulfonate monooxygenase (*ssuE*), all involved in fluorinated compound degradation. Cultivated for 7 days in a liquid medium with C7-C10 PFCAs as the sole carbon and energy source, the strain completely degraded these acids. LC-MS analysis showed that the transformation takes place due to PFHxA with the release of various levels of stoichiometry (depending on the PFCA) of fluorine ion mineralization indicators. Therefore, *Pseudomonas mosselii strain* 5(3) shows genetically confirmed high potential for decomposing C7-C10 PFCAs.

The article “The Association of Perfluoroalkyl Substance Exposure and a Serum Liver Function Marker in Korean Adults” investigated PFAS exposure in Koreans and its relationship with liver function markers (AST, ALT, and GGT). Based on the Korean National Environmental Health Survey (KoNEHS) 2018–2020 (Cycle 4), data from 2961 subjects were analyzed. The geometric mean concentration of PFAS in Korean adults was significantly higher than that in American adults from the National Health and Nutrition Examination Survey (NHANES, 2017–2018). A multivariable linear regression analysis, adjusted for age, sex, BMI, smoking status, alcohol intake, and regular exercise, revealed that some PFASs (PFOA, PFOS, PFHxS, PFNA, and PFDeA) were significantly associated with increased liver enzymes. This highlights the need to recognize the threat of PFASs to human health and to discuss regulations and alternatives. Continuous follow-up studies with well-designed cohorts are necessary.

The paper “Insights into the Understanding of Adsorption Behaviors of Legacy and Emerging Per- and Polyfluoroalkyl substances (PFASs) on Various Anion-Exchange Resins” systematically investigated the adsorptive removal of 10 PFASs using four gel and macroreticular anion-exchange resins. The resins’ adsorption capacities were ranked as follows: gel strong base HPR4700 ≈ macroreticular strong base S6368 ≈ macroreticular weak base A111S > gel weak base WA10. The adsorption process likely involved chemical and Henry regime adsorption or reaction control, with intraparticle diffusion as the major removal step. Co-existing fulvic acid and inorganic anions hindered PFAS removal, with WA10 showing the highest inhibition rates of 17% and 71%, respectively. The adsorption capacity of PFBA decreased from 233 μg/g to 194 μg/g and from 233 μg/g to 67 μg/g in the presence of fulvic acid and inorganic anions, respectively. PFASs were more effectively removed by HPR4700, S6368, and A111S in neutral and alkaline environments, while WA10 failed to remove PFASs under alkaline conditions. This study provided theoretical support for removing PFASs from water using various resins.

The review “A Review: Per- and Polyfluoroalkyl Substances-Biological Degradation” summarized recent findings on the bacterial and fungal degradation of PFASs, including the enzymes involved in these processes. Another review, “Per- and Polyfluoroalkyl Substances in Pregnant Women: Maternal Exposure, Placental Transfer, and Relevant Model Simulation”, explored PFAS exposure pathways in pregnant women, the factors influencing placental transfer efficiency, and the mechanisms behind it. It also described simulation analysis approaches using molecular docking and machine learning to understand these mechanisms and highlighted future research priorities. Notably, molecular docking can simulate PFAS binding to proteins during placental transfer, and machine learning can predict placental transfer efficiency. Thus, future research on maternal–fetal transfer mechanisms of PFASs using simulation analysis is essential to understand their health effects on newborns.

Finally, we extend our heartfelt gratitude to the authors, reviewers, and editorial team for their invaluable contributions to this Special Issue. This special issue offers a crucial snapshot of the current knowledge on the occurrence, fate, removal, and effects of PFASs. The research presented emphasizes the complexity of PFAS contamination and underscores the ongoing need for scientific inquiry and technological innovation.
